# Aircraft Wastewater as a Sentinel for Transboundary Antimicrobial Resistance: An Integrated Genomic Approach

**DOI:** 10.3390/microorganisms14091988

**Published:** 2026-09-08

**Authors:** Abiola Senok, Rashed Alghafri, Douha Shouqair, Subham Verma, Lobna Mohammed, Mohammed Naji, Abdulla Albastaki, Rania Nassar, Dean Everett, Richard Goering, Mushtaq Khan

**Affiliations:** 1 College of Medicine, Mohammed Bin Rashid University of Medicine and Health Sciences, Dubai P.O. Box 505055, United Arab Emirates; 2 Center for Microbial Sciences, Mohammed Bin Rashid University of Medicine and Health Sciences, Dubai P.O. Box 505055, United Arab Emirates; 3 School of Dentistry, Cardiff University, Cardiff CF14 4XY, UK; 4 General Department of Forensic Science and Criminology, Dubai Police, Dubai P.O. Box 1493, United Arab Emirates; 5 Department of Public Health and Epidemiology, College of Medicine and Health Sciences, Khalifa University, Abu Dhabi P.O. Box 127788, United Arab Emirates; 6 Biotechnology Center, Khalifa University, Abu Dhabi P.O. Box 127788, United Arab Emirates; 7 Infection Research Unit, Khalifa University, Abu Dhabi P.O. Box 127788, United Arab Emirates; 8 Department of Medical Microbiology and Immunology, Creighton University School of Medicine, Omaha, NE 68178, USA; 9 Department of Medical Microbiology and Immunology, College of Medicine and Health Sciences, United Arab Emirates University, Al-Ain P.O. Box 15551, United Arab Emirates; 10 Zayed Bin Sultan Center for Health Sciences, United Arab Emirates University, Al-Ain P.O. Box 15551, United Arab Emirates

**Keywords:** aircraft wastewater, antimicrobial resistance, metagenome-assembled genomes, transboundary surveillance

## Abstract

Aircraft wastewater (AWW) provides a composite environmental matrix reflecting passengers from diverse geographic origins and may serve as a surveillance tool for AMR monitoring. To characterize microbial community composition, antimicrobial resistance genes (ARGs), virulence factor genes (VFGs), and genome-resolved features of AWW. Samples (*N* = 10) were collected from long-haul flights arriving in the UAE between October 2024 and February 2025. Shotgun metagenomic sequencing and high-throughput quantitative PCR (HT-qPCR) were performed. Metagenome-assembled genomes (MAGs) were reconstructed. Shotgun metagenomics identified 752 bacterial species dominated by gut-associated families, including Lachnospiraceae and Ruminococcaceae. A total of 345 ARGs were detected, with tetracycline resistance genes being most abundant. Regional variation in resistome composition was supported by permutational multivariate analysis of variance (PERMANOVA, *p* = 0.010), and principal coordinate analysis indicated separation by flight-origin region. MAG reconstruction recovered 1012 genomes, with 85.4% resolved to species level. MAG-based annotation identified 280 VFGs corresponding to 116 non-redundant virulence genes. HT-qPCR confirmed the widespread presence of key ARG classes and mobile genetic elements across samples, in keeping with metagenomic findings. AWW contains diverse microbial communities and AMR determinants. Genome-resolved analysis provided organism-level context for resistome and virulome characterization, confirming AWW as an environmental matrix for monitoring transboundary AMR dynamics.

## 1. Introduction

Antimicrobial resistance (AMR) poses a growing global health threat, weakening the effectiveness of antimicrobial therapies and driving increasing morbidity, mortality, and healthcare costs [1]. With millions of individuals crossing borders daily, international travel accelerates the movement of resistant pathogens and antimicrobial resistance genes (ARGs) across regions. International airports and the aircraft connecting them are increasingly being recognized as potential nodes within global AMR transmission pathways. Aircraft wastewater (AWW), generated from pooled human feces during flights, offers a minimally diluted and temporally discrete snapshot of the microbiome and resistome profiles associated with diverse, highly mobile passenger populations [2,3].

Wastewater-based surveillance (WBS) has become an important approach for population-level monitoring of infectious disease pathogens and AMR. Municipal WBS studies have contributed significantly to understanding population-level resistance patterns [4]. Extending this concept to transport hubs provides a means to identify microbial and genetic signatures introduced via international travel at the point of arrival, before they merge with broader municipal waste streams. The value of AWW for monitoring infectious agents was highlighted during the COVID-19 pandemic, when SARS-CoV-2 and emerging variants were successfully identified in AWW samples [5,6,7].

Recent studies show that AWW contains a diverse repertoire of ARGs. Heß et al. [2] identified high abundances of mobile ARGs in AWW compared with municipal wastewater, suggesting that AWW retains resistance signatures linked to the geographical origins of travelers. Also, Smith et al. [8] demonstrated that ARG detection remains feasible despite the presence of chemical disinfectants used in aircraft sanitation systems. More recent genomic analyses have identified clinically important resistance genes including *bla*_NDM-1_, *bla*_CTX-M_, *ermB*, *qnrS*, *sul1,* and *tetM*, with profiles reflecting regional AMR prevalence patterns [3]. Additionally, comparisons between AWW, airport terminal wastewater, and municipal influent have shown distinct microbial and resistome compositions, highlighting the potential of AWW to capture signals linked to flight origin [9]. Despite these developments, AWW-based AMR surveillance remains limited in the Middle East, a region that hosts some of the world’s busiest transit hubs and links populations with widely varying AMR burdens.

Shotgun metagenomic sequencing provides comprehensive, untargeted characterization of microbial communities, ARGs, and mobile genetic elements, while high-throughput quantitative PCR enables sensitive, absolute quantification of targeted ARGs. Combining both approaches can strengthen interpretation and enhance detection of low-abundance resistance markers within complex matrices such as AWW. However, the integration of shotgun metagenomics with high-throughput qPCR in AWW studies remains limited [9]. In this study, we conducted systematic sampling of aircraft wastewater in Dubai, United Arab Emirates (UAE), a major international aviation hub connecting regions with heterogeneous AMR burdens. Using combined shotgun metagenomic sequencing and high-throughput qPCR, we characterized the microbial communities and ARG profiles present in AWW. We aimed to describe the taxonomic composition and diversity of AWW microbiota; quantify the abundance and diversity of ARGs using complementary sequencing- and qPCR-based approaches; and assess the potential of AWW as a scalable surveillance tool for monitoring cross-border AMR dissemination. This work contributes new data from a major Middle Eastern transit hub and advances the application of integrated molecular methods for AMR surveillance in aircraft wastewater.

## 2. Materials and Methods

### 2.1. Samples Collection

AWW samples were collected at Dubai International Airport, UAE, between October 2024 and February 2025. The sampling focused on incoming long-haul international flights, with two aircraft selected each month using a randomized schedule. Wastewater was obtained immediately upon aircraft arrival during lavatory tank servicing, following aseptic procedures. From each aircraft, 1000 mL of wastewater was collected directly into sterile polypropylene containers. The wastewater samples were placed immediately in temperature-controlled chiller boxes maintained at 4 °C and transported to the laboratory for processing on the same day. In the laboratory, samples were homogenized by gentle inversion, and coarse debris was removed by allowing the sample to rest. The clarified supernatant was used for downstream work.

### 2.2. High-Throughput Quantitative PCR (HT-qPCR)

Total DNA was extracted using the DNeasy PowerWater Kit (QIAGEN, Hilden, Germany) following manufacturer instructions, with optimizations for AWW. AWW was passed through a 5 mm diameter polyethersulfone (PES) membrane filter with a pore size of 0.22 µm to capture microbial biomass for DNA extraction. Following filtration, each membrane was aseptically collected using a sterile scalpel and forceps, folded, and transferred directly into the lysis tube. DNA was subsequently extracted according to the manufacturer’s instructions. DNA purity and concentration were assessed using NanoDrop spectrophotometry (Thermo Fisher Scientific, Waltham, MA, USA) [10].

DNA purity and concentration were assessed using NanoDrop spectrophotometry [10]. Extracted DNA was then stored at −80 °C for downstream analysis. High-throughput qPCR analysis was performed using the Resistomap platform (Resistomap Oy, Helsinki, Finland), comprising 72 primer sets targeting antimicrobial resistance genes (ARGs), mobile genetic elements (MGEs), taxonomic markers, and the 16S rRNA gene for normalization (Supplementary Data S1). The primer sets were selected from the validated SmartChip 96 assay One Health panel (Resistomap, Finland). The SmartChip™ Real-Time PCR system (Takara Bio, San Jose, CA, USA) was used, and assays were performed according to Resistomap’s established workflow [10,11,12,13]. Briefly, cycling conditions included an initial denaturation at 95 °C for 10 min, followed by 40 cycles of denaturation at 95 °C for 30 s and annealing/extension at 60 °C for 30 s [10,11,12,13]. Melting curve analysis was performed for each primer set, and assays showing non-specific amplification or multiple peaks were excluded from further analysis. Each sample was analyzed in technical triplicate, and a no-template control was included in every qPCR run. A gene was deemed as detected if amplification occurred in at least two out of three replicates, after which the mean cycle threshold (Ct) value was calculated. A Ct threshold of 27 was used as the limit of detection, consistent with validated Resistomap protocols for environmental and wastewater samples [10,11,12,13]. Relative gene abundance was calculated using the 2^−ΔCt^ method, where ΔCt = Ct(target gene) − Ct(16S rRNA), enabling comparison across samples and gene classes. Gene copy numbers were normalized to 16S rRNA copy number to account for variation in microbial biomass.

### 2.3. Shotgun Metagenomic Sequencing

Total community DNA was extracted using the DNeasy PowerSoil Pro Kit (QIAGEN, Hilden, Germany) according to the manufacturer’s instructions. Purified DNA was washed, eluted, and quantified using a Qubit Flex fluorometer and the Qubit dsDNA High Sensitivity Assay Kit (Thermo Fisher Scientific, Waltham, MA, USA) [14,15,16].

DNA libraries were prepared with the Watchmaker DNA Library Prep Kit with Fragmentation (7K0019-1K; Watchmaker Genomics, Boulder, CO, USA), following the validated CosmosID workflow (CosmosID, Germantown, MD, USA) [14,15,16]. Briefly, 1 ng of genomic DNA was subjected to enzymatic fragmentation, end repair, and A-tailing using a master mix containing Watchmaker Frag/AT Buffer and Frag/AT Enzyme Mix, with incubation at 37 °C for 5 min and subsequently at 65 °C for 30 min. IDT xGen Stubby Adapters were then ligated to the processed DNA using the kit-provided ligation reagents at 20 °C for 15 min. The adapter-ligated libraries were purified with CleanNGS magnetic beads (CleanNA, Waddinxveen, The Netherlands) and eluted in 10 mM Tris-HCl (pH 8.0). IDT xGen UDI Primers and the kit-provided amplification mix were subsequently added, followed by amplification of the libraries for sixteen PCR cycles. Amplified libraries were purified with CleanNGS magnetic beads and eluted in nuclease-free water. Final library concentrations were determined using the Qubit fluorometer with the dsDNA High Sensitivity Assay (Thermo Fisher Scientific). Libraries were converted for platform compatibility using the Adept workflow (Element Biosciences, San Diego, CA, USA) [14,15,16]. Paired-end sequencing (2 × 150 bp) was performed on the Element AVITI system using Cloudbreak sequencing chemistry (Element Biosciences, San Diego, CA, USA) [14,15,16,17].

### 2.4. Bioinformatic Analysis

Microbial taxonomic composition, antimicrobial resistance (AMR), and virulence factor (VF) profiles were determined using the CosmosID (v2.0; Rockville, MD, USA) metagenomic bioinformatics platform (https://cosmos-hub.com/, accessed on 30 August 2026). Taxonomic profiling was performed using the CosmosID Taxa–Kepler Domain workflow (pipeline v1.1.0, database v3.0.0), a k-mer-based, host-agnostic workflow optimized for complex environmental and wastewater metagenomes that queries quality-filtered reads against curated reference databases using variable-length, species- and strain-specific k-mer signatures, enabling taxonomic resolution to species and, where supported, strain level. Reads without a matching microbial k-mer signature, including host-derived reads, are not assigned and do not contribute to reported relative abundances. Relative abundance was calculated as reads per million and normalized to total prokaryotic reads. AMR genes and VFs were identified using the ResFinder database (https://cge.food.dtu.dk/services/ResFinder/, accessed on 30 August 2026) and VFDB (VFDB: Virulence Factor Database) via the CosmosID Kepler-AMR/VF Profiler (pipeline v1.1.0, database v1.1.0), which uses hierarchical k-mer indexing of curated nucleotide sequences, with abundance estimated from composite k-mer coverage statistics and reported as relative abundance scores.

Assembly-based analysis: Raw sequencing reads were processed using the nf-core/mag pipeline. Adapter and quality trimming were performed with fastp, retaining reads with a minimum quality score of Q15 and a minimum length of 15 bp. Quality-filtered reads were assembled using MEGAHIT v1.2.9 (https://github.com/voutcn/megahit, accessed on 30 August 2026), retaining contigs ≥1500 bp for binning. Metagenome-assembled genomes (MAGs) were reconstructed using MetaBAT 2 v2.17 (https://bitbucket.org/berkeleylab/metabat, last accessed on 30 August 2026) following read alignment to contigs with Bowtie2 v2.4.2 (http://bowtie-bio.sourceforge.net/bowtie2, accessed on 30 August 2026) and BAM file processing using SAMtools v1.11 (http://www.htslib.org, accessed on 30 August 2026). MAG quality was assessed with CheckM v1.2.3 (https://ecogenomics.github.io/CheckM, accessed on 30 August 2026), retaining bins with ≥50% completeness and ≤10% contamination. MAG taxonomic classification was performed using GTDB-Tk v2.4.1 (https://ecogenomics.github.io/GTDBTk, accessed on 30 August 2026) against GTDB Release 226, using a minimum alignment fraction of 0.65 and a minimum percentage of amino acids in the MSA of 10%. Genome annotation was conducted with Bakta v1.10.4 (https://github.com/oschwengers/bakta, accessed on 30 August 2026). ARGs were identified using CARD (https://card.mcmaster.ca, accessed on 30 August 2026) via RGI v6.0.5 (https://github.com/arpcard/rgi, accessed on 30 August 2026), and virulence factor genes (VFGs) were detected using VFDB with ABRicate v1.2.0 (https://github.com/tseemann/abricate, accessed on 30 August 2026) using the tool’s default minimum thresholds of 80% sequence identity and 80% coverage.

Statistical analysis: Alpha diversity (Shannon index) was calculated within the CosmosID platform (https://cosmos-hub.com/, accessed on 30 August 2026) and compared across flight-origin cohorts using the Wilcoxon rank-sum test. Beta diversity (Bray–Curtis dissimilarity) was assessed by principal coordinate analysis (PCoA), with statistical significance of community-level clustering by flight-origin region determined using permutational multivariate analysis of variance (PERMANOVA) at 999 permutations. The same PERMANOVA approach was applied to resistome composition (antibiotic-class profiles) to test for regional structuring. Co-occurrence relationships among the 40 most abundant bacterial genera, ARGs, and VFGs were assessed by Spearman’s rank correlation, computed in Python (v3.9.6; SciPy v1.13.1 spearmanr), using genus- and gene-level abundance matrices derived from the RGI (CARD database v4.0.1) and ABRicate (VFDB database v1.2.0) outputs. To prioritize strong, high-confidence associations and limit spurious edges, a stringent threshold (|*ρ*| > 0.7, *p* < 0.01) was applied, consistent with conventional cutoffs used in microbiome and AMR co-occurrence network studies. Correlation pairs were additionally required to have non-zero abundance in at least five of the ten aircraft wastewater samples, ensuring sufficient paired observations for stable correlation estimation. Retained pairs were visualized as a correlation network using the Python NetworkX v3.2.1 package.

## 3. Results

### 3.1. Quantitative PCR Profiling of Taxonomic Markers, ARGs, and MGEs

**Figure 1:**
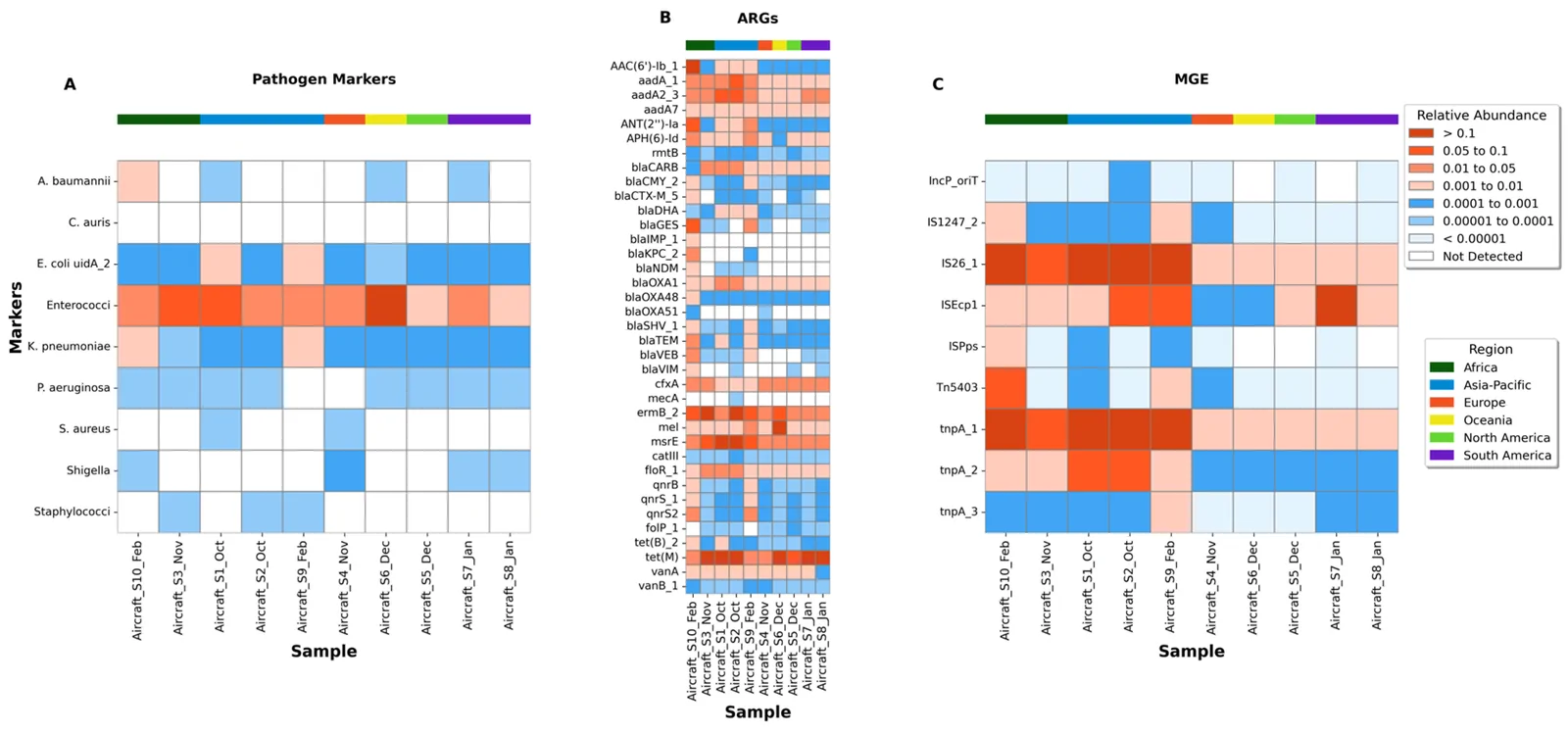
Distribution of pathogen markers, antimicrobial resistance genes, and mobile genetic elements across aircraft samples. Heatmaps showing relative abundance of (**A**) pathogen-specific markers, (**B**) antimicrobial resistance genes (ARGs) by drug class, and (**C**) mobile genetic elements (MGEs) across 10 aircraft samples from six global regions. Color intensity represents relative abundance from white (not detected) to dark red-brown (>0.1). Colored bars above heatmaps indicate sample origin: Africa (dark green), Asia-Pacific (blue), Europe (red), North America (light green), Oceania (yellow), and South America (purple).

The detection rate was high, with 57–65 of the 72 gene targets identified in each sample. The highest number of detectable targets was observed in wastewater from flights that originated from Africa (65 genes), followed by those from the Asia-Pacific region (62–63 genes). Wastewater from North America (58 genes) and Oceania (57 genes) flights exhibited slightly lower diversity. Pathogen-associated and taxonomic markers highlighted the widespread presence of clinically relevant bacteria in aircraft wastewater. Markers for *Escherichia coli*, *Klebsiella pneumoniae*, and *Enterococcus* spp. were detected in all samples, with higher relative abundance observed in Asia-Pacific, African, and Oceania originating flight samples, respectively. *Pseudomonas aeruginosa* was detected in most samples (*n*/*N* = 8/10), whereas the occurrence of *Acinetobacter baumannii* was notably lower (*n*/*N* = 4/10). Markers for *Shigella* spp., *Staphylococcus* spp., and *Staphylococcus aureus* were detected in smaller subsets of samples, whilst *Candida auris* was not identified in any sample (Figure 1A).

#### Resistome and Mobilome

Resistance determinants for aminoglycosides, β-lactams, macrolide–lincosamide–streptogramin B (MLSB), tetracyclines, quinolones, and vancomycin were present in all samples irrespective of flight origin. Similarly, all screened MGEs were detected across samples. Although these findings demonstrated the occurrence of the ARG–MGE signature across all samples, important patterns of regional variation in relative abundances were observed. The occurrence of carbapenemase markers varied by flight origin, with *bla*_IMP-1_, *bla*_KPC-2,_ and *bla*_NDM_ being detected only in wastewater from flights originating in Africa and Asia-Pacific (Figure 1B). Additionally, samples from these flights also exhibited higher relative abundance of *bla*_OXA-48_, *bla*_TEM_, *bla*_CMY-2_, and *bla*_SHV-1_ compared to other regions. In contrast, samples from Asia-Pacific region flights showed relatively elevated abundance of *bla*_CARB_, *bla*_DHA_, and *bla*_OXA-1_ (Figure 1B). The relative abundance for other beta-lactamase genes, notably *bla*_CTX-M5_, *bla*_GES_, and *bla*_VIM,_ was heterogeneous across regions (Figure 1B). MGEs demonstrated similar regional structuring, with higher relative abundance of *IS26*, *tnpA_1*, and *tnpA_3* seen in African and Asia-Pacific samples (Figure 1C). The highest relative abundance of *ISEcp1* was observed in wastewater from South American flights, while the Asia-Pacific samples showed the highest relative abundance of *tnpA_2*. These patterns indicate regional differences not only in ARG composition but also in the MGEs associated with resistance dissemination (Figure 1C).

### 3.2. Shotgun Metagenomic Profiling of Aircraft Wastewater

#### 3.2.1. Microbial Community Composition

**Figure 2:**
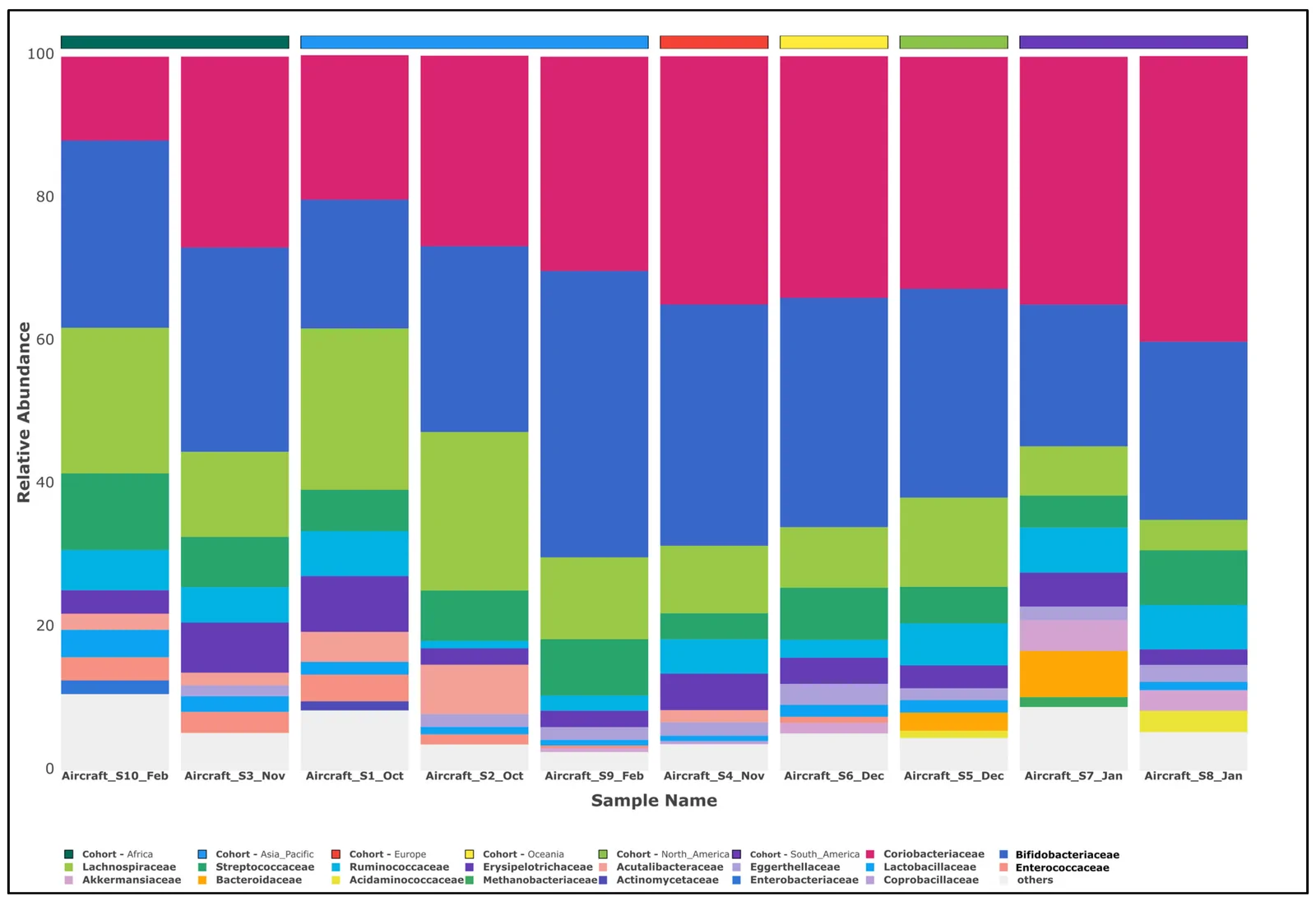
Family-level composition of aircraft wastewater metagenomes by flight origin. Ten aircraft wastewater metagenomes from six geographic regions (Africa, Asia-Pacific, Europe, North America, South America, and Oceania) were analyzed. Stacked bars show relative abundance (%) of dominant bacterial families per sample; each column represents one aircraft, and the colored strip above indicates flight origin.

The ten aircraft wastewater metagenomes enabled characterization of microbial community composition across the six flight-origin regions (Supplementary Table S1). Taxonomic assignment identified 752 bacterial species across all samples, with communities enriched for *Coriobacteriaceae*, *Bifidobacteriaceae*, *Lachnospiraceae*, *Streptococcaceae*, and *Ruminococcaceae*. The ten most abundant species were detected across regions, representing 17 dominant families. Family-level relative abundance patterns varied between flights (Figure 2). Alpha diversity was consistently high across samples (Shannon index: 4.33–4.86), with comparable within-sample microbial richness across flight-origin regions. Clinically important *Enterobacterales*, including *E. coli*, *K. pneumoniae*, *K. quasipneumoniae*, *Citrobacter freundii*, *Salmonella enterica*, and *Enterobacter* spp., were detected across samples. Although several ESKAPE-associated taxa were identified, *A. baumannii* and *P. aeruginosa* were not detected. Low-abundance non-bacterial taxa were detected, including protists (*Cyclospora cayetanensis*, *Reticulomyxa filosa*) and the opportunistic yeast *C. glabrata*.

#### 3.2.2. Resistome and Virulome Profile

**Figure 3:**
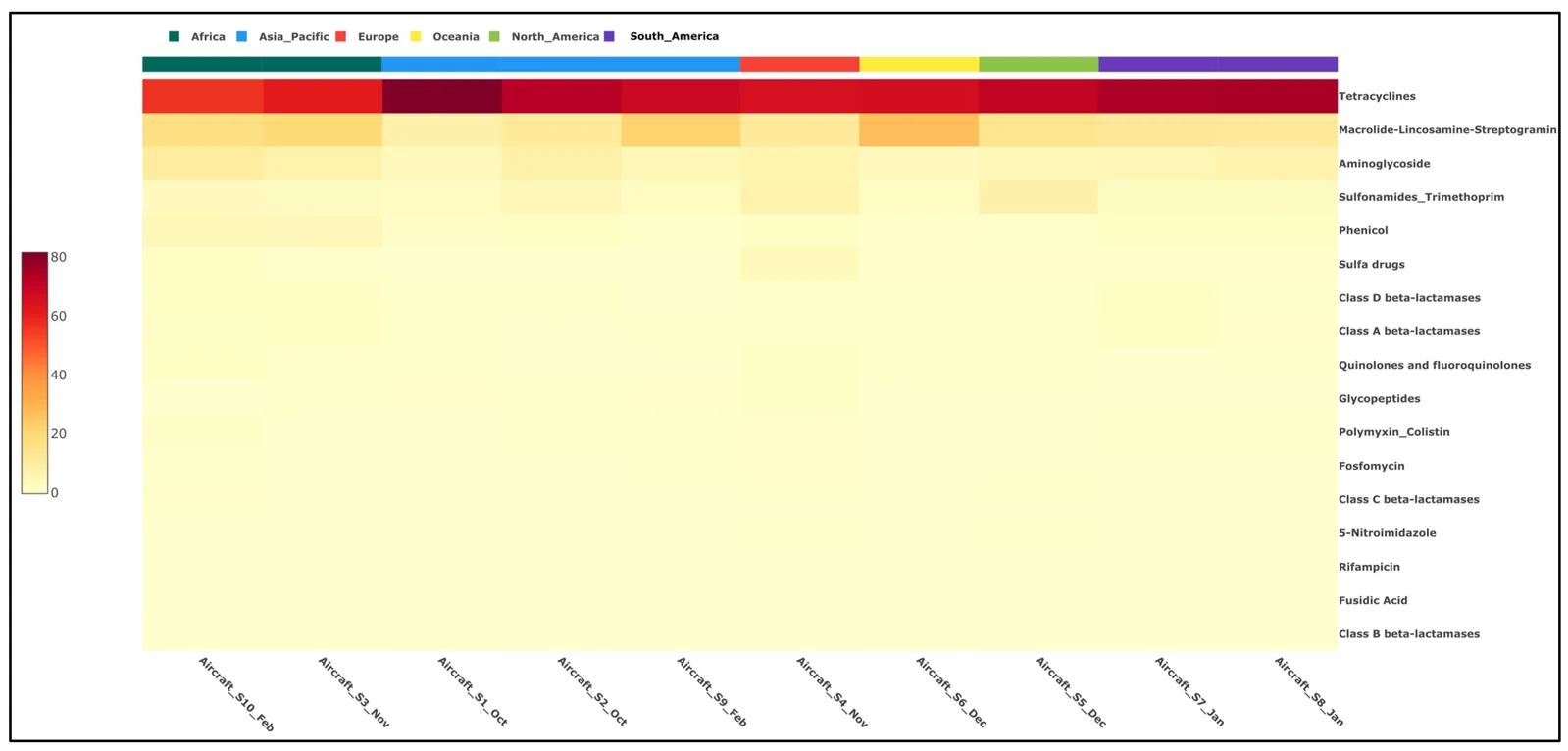
Regional clustering based on antibiotic-class resistome profiles. Hierarchical clustering heatmap showing relative abundance of AMR determinants grouped by antibiotic class across aircraft wastewater metagenomes. Columns represent individual aircraft samples, and rows correspond to antibiotic classes. The colored bar above the dendrogram indicates flight-origin region (Africa, Asia-Pacific, Europe, North America, South America, and Oceania). Warmer colors denote higher relative abundance. Tetracycline and macrolide–lincosamide–streptogramin resistance dominated across samples, while aminoglycoside and sulfonamide–trimethoprim determinants were variably distributed. Samples clustered according to resistome composition, revealing partial geographic structuring of AMR profiles across regions.

Across the ten AWW metagenomes, 345 ARGs were detected, with resistome composition showing statistically significant variation across flight-origin regions (PERMANOVA, *p* = 0.010). Regional clustering based on antibiotic-class composition is shown in Figure 3. Flights originating from Europe (176 unique ARGs) and Africa (173 unique ARGs) exhibited the greatest within-cohort ARG richness. Across all samples, tetracycline resistance genes predominated, including *tetM*, *tet41*, *tet44*, *tetC*, and *tetG*. High-frequency ARGs from additional antimicrobial classes were detected, including macrolide–lincosamide–streptogramin associated resistance genes (*mefA*, *msrD*, *msrE*, *mph(E)*, *erm*, *lnu*, *lsa*), folate-pathway resistance genes (*dfrF*, *dfrE*, *sul1/2/3*), multiple β-lactamase genes (*bla*_TEM_, *bla*_CARB_, *bla*_GES_, *bla*_VEB_, *bla*_AmpC_, *bla*_CMY_, *bla*_DHA_, *bla*_OXA_), and determinants associated with quinolone, colistin, and glycopeptide resistance (*qnr*/*qxAB*, *pmr*, *vanA/B*). PCoA supported the regional structuring of resistome profiles (PC1 = 40.3% variance; PERMANOVA, *p* = 0.010).

**Figure 4:**
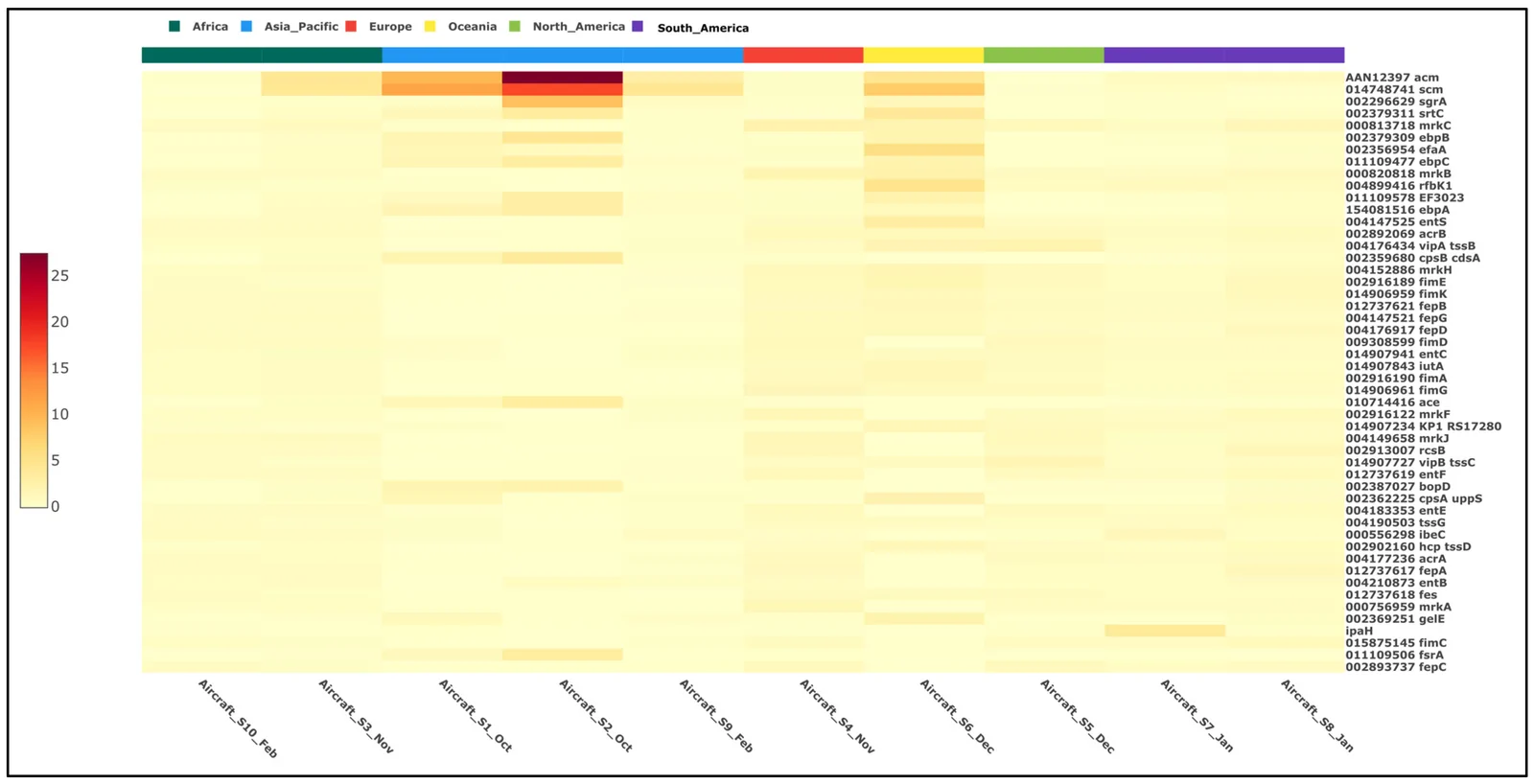
Distribution of predominant virulence factor genes across aircraft wastewater samples. Heatmap showing relative abundance of detected virulence factor genes (VFGs) across aircraft samples. Columns represent individual aircraft, and rows correspond to annotated virulence genes. The colored strip above indicates flight-origin region (Africa, Asia-Pacific, Europe, North America, South America, and Oceania). Warmer colors denote higher relative abundance. Core colonization and fitness-associated determinants, including adhesion, iron acquisition, secretion system, and stress response genes, were broadly conserved across samples, while several genes displayed region-specific enrichment, indicating heterogeneous carriage of virulence traits among passenger-associated microbial communities.

**Figure 5:**
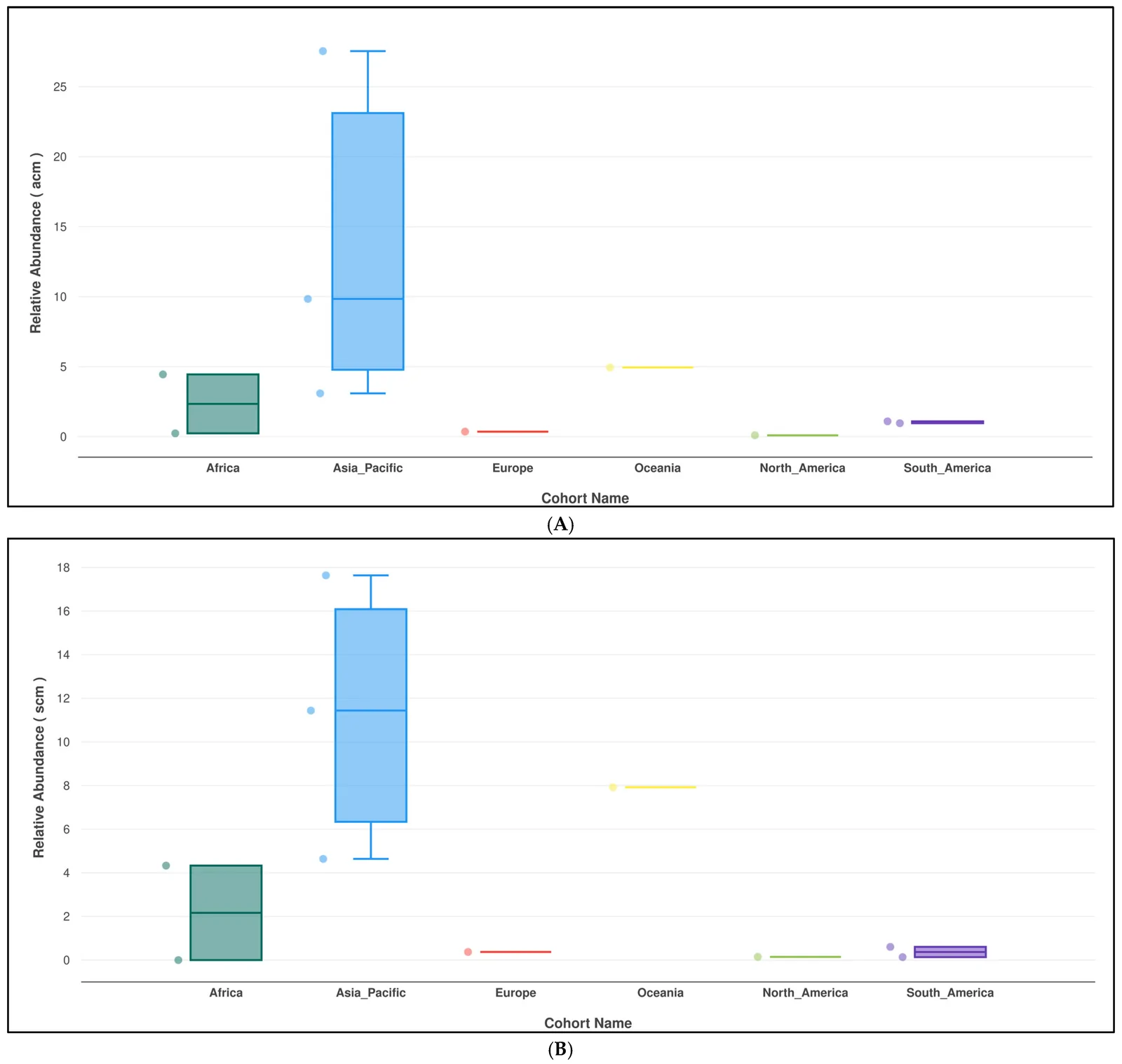
Relative abundance distribution of *acm* and *scm* virulence genes. Boxplots showing the relative abundance of (**A**) *acm* and (**B**) *scm*, (collagen adhesin genes characteristically associated with *Enterococcus faecium* and *Enterococcus faecalis,* respectively), in aircraft wastewater metagenomes stratified by flight-origin region (Africa, *n* = 2; Asia-Pacific, *n* = 3; Europe, *n* = 1; North America, *n* = 1; South America, *n* = 2; Oceania, *n* = 1).

Virulome profiling identified 540 VFGs across samples, with predominant VFGs shown in Figure 4. Virulence determinants detected across all samples included *Enterococcus* spp.-associated genes *asa1*, *gelE*, and *esp*, as well as siderophore-related genes (*rmpA*, *iuc*, and *iut*). In contrast, *Enterococcus* spp. adhesion-associated markers exhibited regional variation, with samples from the Asia-Pacific cohort showing higher relative abundances of *acm* (~0.275) and *scm* (~0.176) (Figure 5A and Figure 5B, respectively).

### 3.3. Assembly and Taxonomic Assignment from Metagenomes

A total of 1012 metagenome-assembled genomes (MAGs) were recovered from the ten AWW metagenomes. Based on MIMAG quality criteria, 222 MAGs (21.9%) were classified as high quality (≥90% completeness, ≤5% contamination), and 342 MAGs (33.8%) as medium quality (≥50% completeness, ≤10% contamination). In total, 564 MAGs (55.7%) met medium- or high-quality thresholds. MAG recovery varied among individual samples, with the number of MAGs per sample ranging from 13 (Aircraft_S6, Oceania) to 168 (Aircraft_S3, Africa). Across samples, high-quality MAGs represented 15.4–30.9% of recovered genomes, while medium-quality MAGs accounted for 26.6–41.3%. The mean MAG completeness per sample ranged from 53.9% to 67.1%, and the mean contamination remained low (1.6–3.8%) across all metagenomes (Table 1). Among the 564 medium- and high-quality MAGs, genome sizes ranged from 0.46 to 5.00 Mbp (mean 2.03 Mbp), with an average of 343.2 contigs per MAG (range: 8–1363). When aggregated by flight-origin region, MAG recovery was broadly consistent, with 53.6–57.4% of MAGs per region meeting medium- or high-quality thresholds, except for Oceania, which exhibited lower recovery likely attributable to reduced sequencing depth rather than biological differences. High-quality MAG proportions were remarkably stable across major regions (~20–25%), indicating reproducible genome recovery independent of geographic origin and supporting the robustness of genome-resolved analysis across samples (Supplementary Table S2).

**Figure 6:**
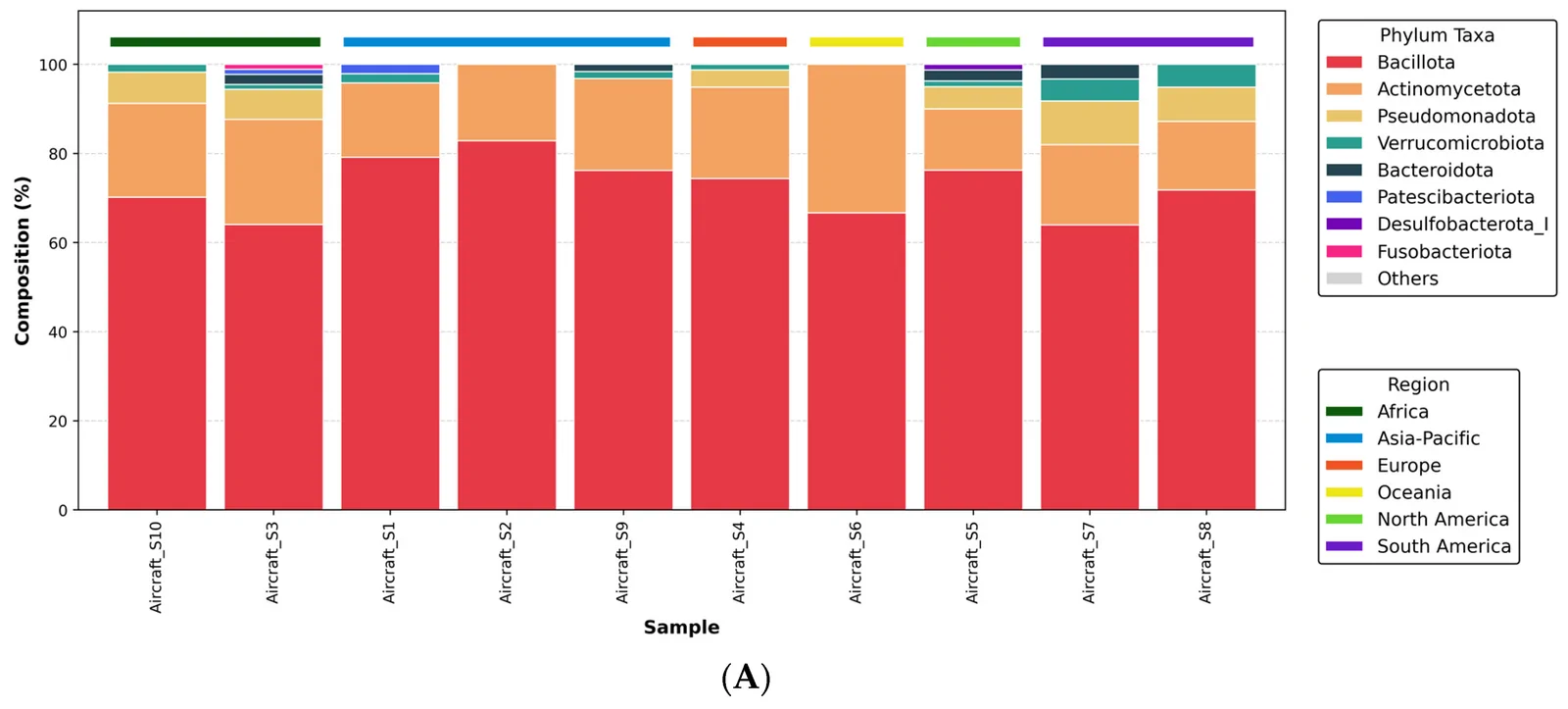

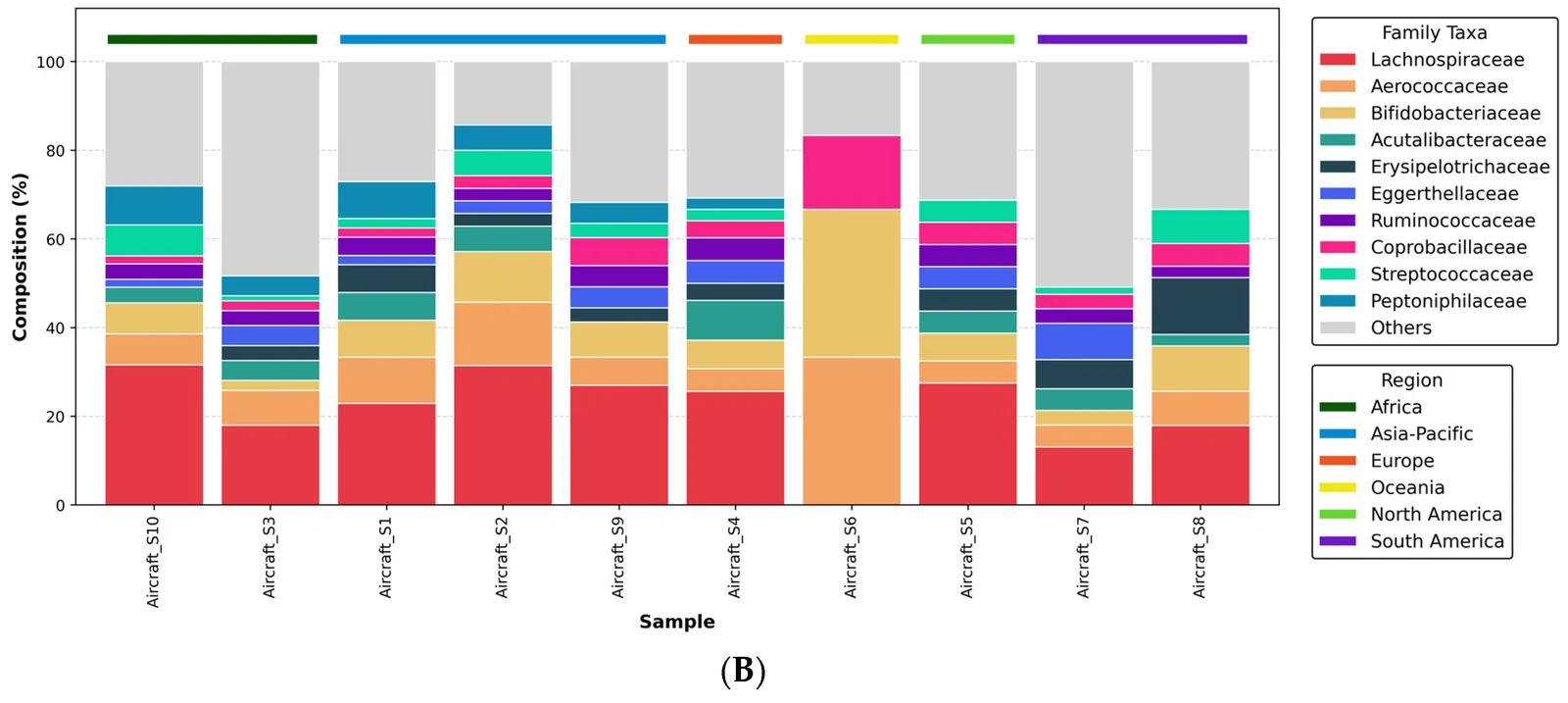

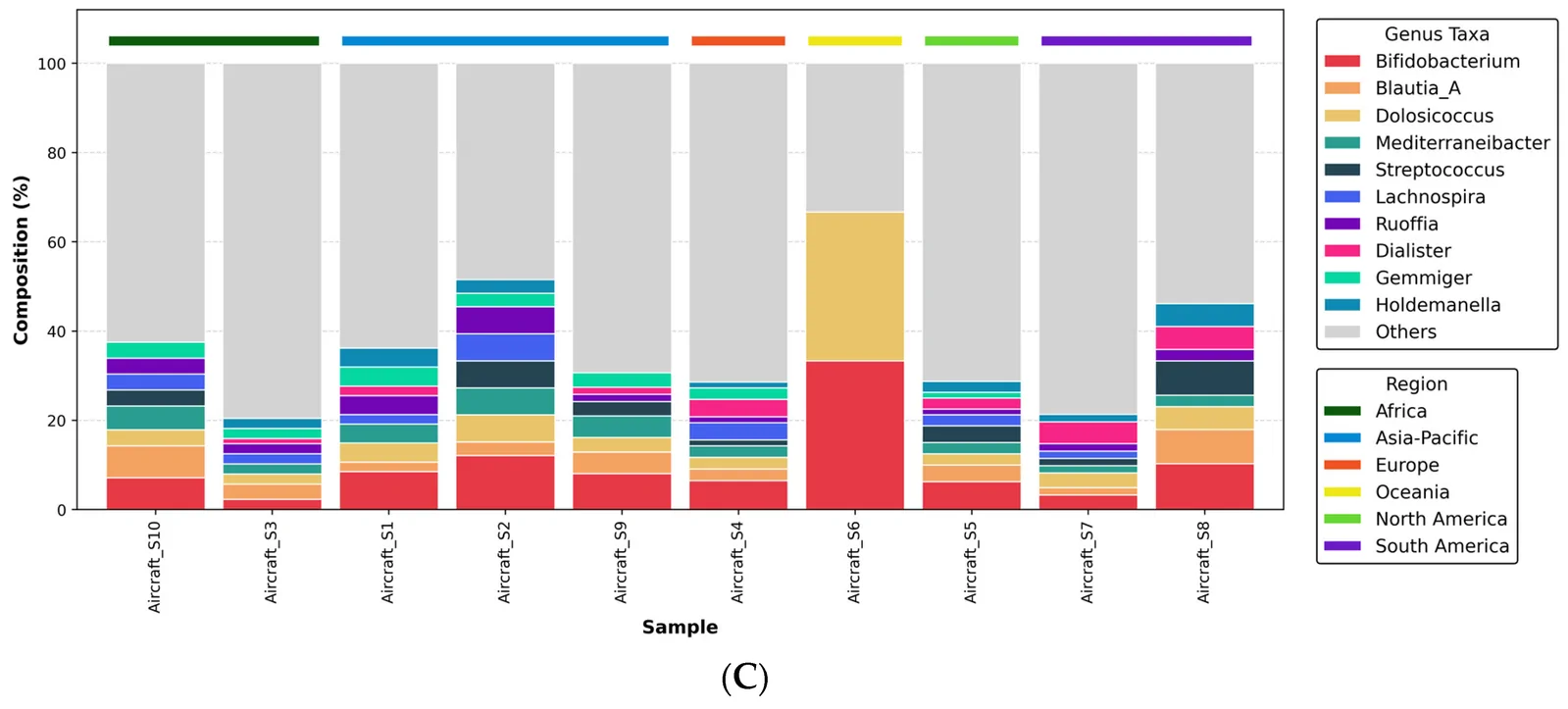

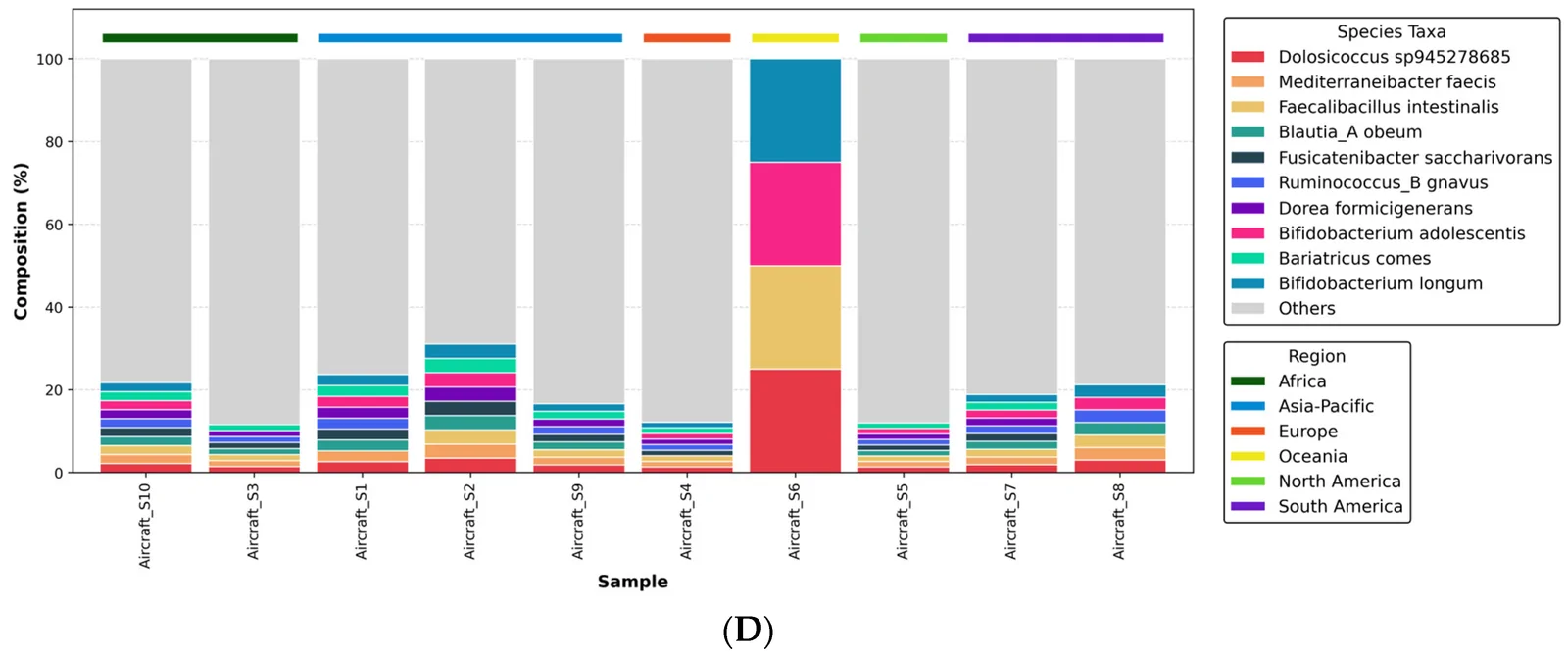
Taxonomic composition of metagenome-assembled genomes (MAGs). Stacked bar plots showing the relative composition (%) of the top ten taxa across reconstructed MAGs at multiple taxonomic levels: (**A**) phylum, (**B**) family, (**C**) genus, and (**D**) species. Each column represents an individual aircraft sample, and colors denote taxa.

Of the 564 high- and medium-quality MAGs recovered, 556 (98.6%) were assigned a taxonomic lineage using GTDB-Tk. Taxonomic resolution was high across ranks, with all classified MAGs assigned at the family level (100%), 549 MAGs (98.7%) resolved to the genus level, and 475 MAGs (85.4%) resolved to the species level. At the phylum level, aircraft wastewater microbiomes were dominated by *Bacillota* (~72% of HQ+MQ MAGs), followed by *Actinomycetota* (~19%) and *Pseudomonadota* (~5%), with minor contributions from *Verrucomicrobiota*, *Bacteroidota*, and other low-abundance phyla (Figure 6A). At the family and genus levels, obligate and facultative anaerobic taxa typical of the human gut—including *Lachnospiraceae*, *Ruminococcaceae*, *Bifidobacteriaceae*, *Oscillospiraceae*, *Eggerthellaceae*, and commensal genera such as *Blautia*, *Agathobacter*, *Bifidobacterium*, *Akkermansia*, *Fusicatenibacter*, and *Dorea*—predominated across samples (Figure 6B,C). Clinically relevant genera, including *Enterococcus* and *Streptococcus*, were consistently detected. Species-level analysis further identified gut-associated taxa such as *Faecalibacillus intestinalis*, *Fusicatenibacter saccharivorans*, *Blautia obeum*, and *Ruminococcus gnavus* (Figure 6D). Importantly, *E. coli*, *E. faecium*, and *K. pneumoniae* were recovered as high- or medium-quality MAGs, demonstrating the capacity of aircraft wastewater metagenomics to capture intact genomes of globally relevant taxa within an aggregated community context.

Principal component analysis (PCA) based on the relative abundance of the top 100 bacterial genera revealed partial clustering of aircraft wastewater samples by flight-origin region, with substantial overlap among groups. Consistent with this pattern, PERMANOVA based on Bray–Curtis dissimilarities did not detect statistically significant differences in overall microbial community composition between regions (*p* = 0.092).

### 3.4. ARGs and Virulence Factor Profiles of MAGs

**Figure 7:**
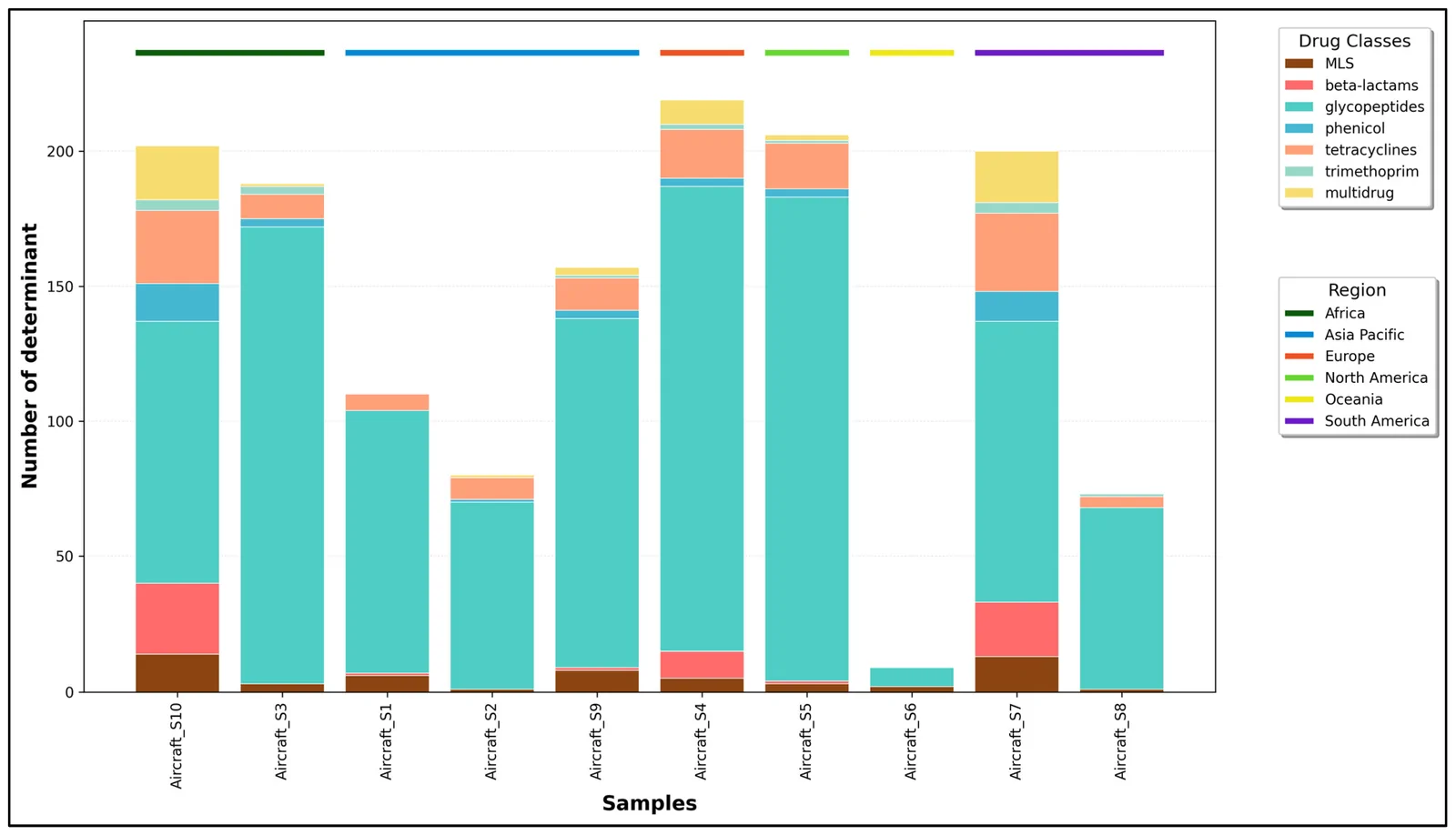
Antimicrobial resistance gene class composition across MAGs. Stacked bar plots showing the resistance drug classes and multidrug resistance identified in metagenome-assembled genomes (MAGs) for each aircraft sample. The colored strip above the bars indicates flight-origin region.

ARG and VFG profiles inferred from MAGs are presented to validate and refine read-level resistome and virulome findings by linking functional determinants to reconstructed genomes, rather than to infer region-specific prevalence or transmission dynamics. Analysis of MAGs using the Resistance Gene Identifier (RGI) against the Comprehensive Antibiotic Resistance Database (CARD) identified a diverse repertoire of ARGs across all aircraft wastewater samples. ARGs spanning multiple antimicrobial classes, including resistance to last-line agents, were detected. While variability in ARG class composition was observed between samples, this heterogeneity primarily reflects differences in community composition and sequencing depth rather than discrete regional patterns. Glycopeptide resistance genes were the most abundant ARGs, driven by widespread detection of *van* operon components (*vanG*, *vanH*, *vanT*, *vanW*, *vanY*, and *vanXY*). Multiple *van* genes were frequently detected within individual samples, consistent with the presence of intact vancomycin resistance gene clusters within the microbial community. β-lactam resistance genes, including those associated with β-lactamases, altered penicillin-binding proteins, and efflux mechanisms, were also detected, alongside ARGs annotated to efflux systems and outer membrane proteins commonly associated with *E. coli* and *K. pneumoniae*. Resistance genes associated with the macrolide–lincosamide–streptogramin (MLS) group and tetracycline resistance were prevalent, with variation in abundance reflecting differences in MAG composition. ARGs conferring resistance to phenicols and trimethoprim were detected at lower but consistent frequencies (Figure 7). Multidrug resistance–associated ARGs were identified in most samples. The full list of MAG-derived ARGs, their associated drug classes/antimicrobial agents, and host bacterial genera/species is provided in Supplementary Data S2.

Across the ten aircraft-derived metagenomic samples, 280 VFGs corresponding to 116 non-redundant virulence genes were identified using VFDB. The VFG repertoire exhibited heterogeneity across samples, with differences in gene carriage reflecting variation in underlying microbial community composition. Detected VFGs included adhesion and colonization factors (e.g., *ompA*, *cblA*), stress-response and regulatory proteins (*hns*, *crp*), and iron acquisition and membrane integrity factors (*arnT*, *lptD*), many of which are multifunctional and commonly detected in gut-associated and wastewater bacteria. Overall, these findings indicate variability in virulence gene carriage across aircraft wastewater samples within the microbial community, rather than differences in bacterial pathogenic potential or disease risk.

### 3.5. Integrated Network Analysis of Taxa, ARGs, and Virulence Factors

**Figure 8:**
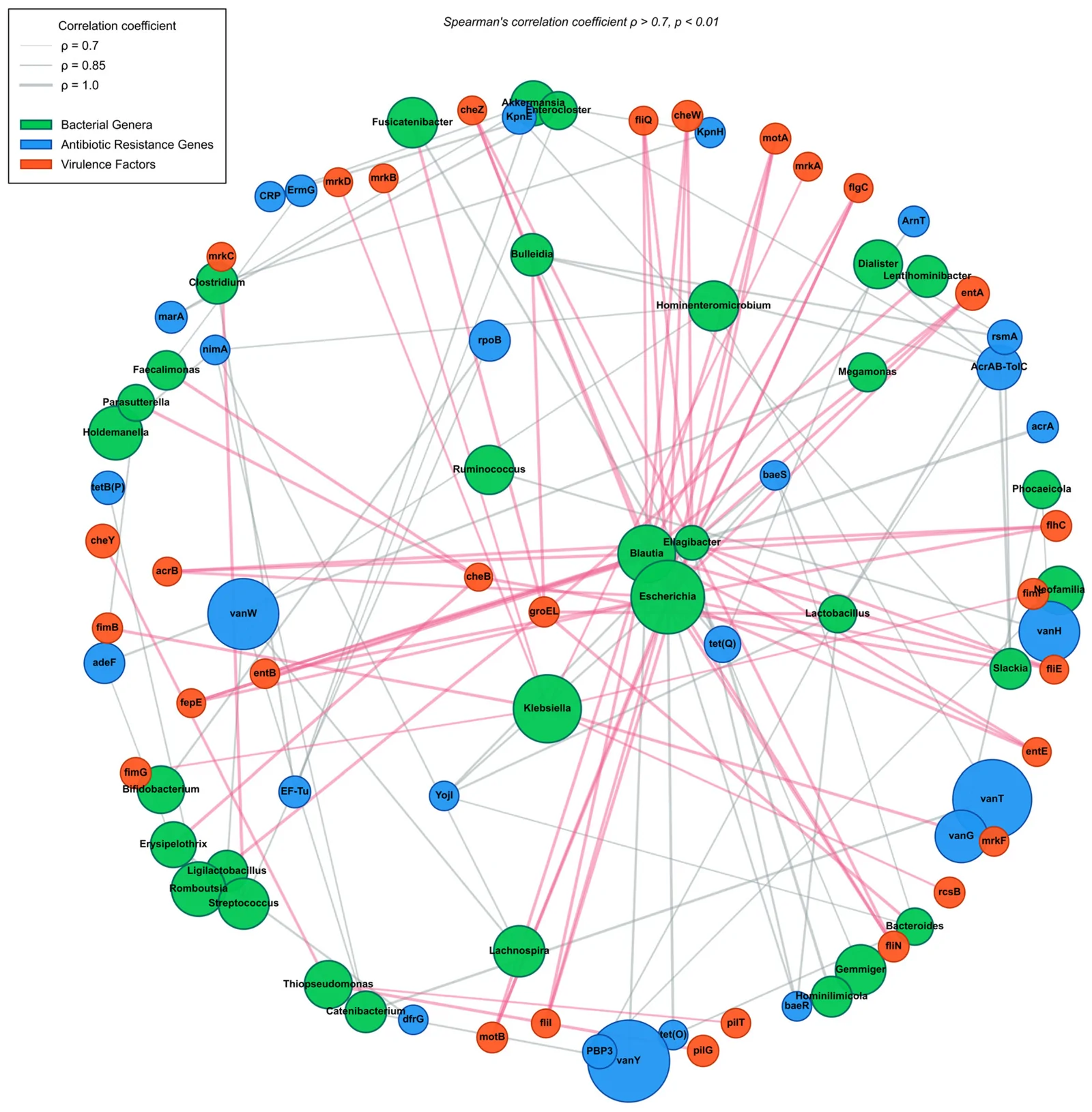
Network analysis showing the relationships between the 40 most abundant antimicrobial resistance genes, bacterial genera, and virulence factors. Correlation network depicting associations among the 40 most abundant antimicrobial resistance genes (ARGs), bacterial genera, and virulence factors detected in aircraft wastewater MAGs. Edges represent strong positive correlations (Spearman’s ρ > 0.7, *p* < 0.01), with grey lines indicating associations between bacterial genera and ARGs and pink lines indicating associations between bacterial genera and virulence factors. Node size corresponds to relative abundance, and edge thickness indicates correlation strength, highlighting co-occurrence patterns between specific taxa and resistance–virulence determinants within the wastewater microbiome.

Network analysis of the top 40 most abundant ARGs and bacterial genera identified 124 statistically significant correlations (Spearman’s ρ > 0.7, *p* < 0.01), highlighting structured co-occurrence patterns between dominant community members, resistance determinants, and virulence-associated genes at the community level (Figure 8). Glycopeptide resistance genes constituted a substantial proportion of the network, with *vanT*, *vanW*, and *vanY* among the most frequently detected ARGs. Gut-associated genera, including *Escherichia* (115 ARGs, 160 VF associations) and *Klebsiella* (76 ARGs, 129 VF associations), emerged as highly connected nodes linking multiple resistance and virulence determinants, reflecting their well-established role as major reservoirs of ARGs in wastewater-associated microbiomes. Strong positive correlations (ρ = 1.000) were also observed between commensal genera (e.g., *Blautia* and *Faecalibacillus*) and iron acquisition–associated genes commonly involved in bacterial fitness and persistence. Notably, the plasmid-encoded *acrD* gene (RND efflux pump) and the *SHV-11* β-lactamase gene were detected in *E. coli* and *K. pneumoniae*, respectively. The detection of plasmid-associated ARGs within *Escherichia* and *Klebsiella* MAGs further highlights the potential mobility of resistance determinants within dominant community members. Overall, the network analysis indicates that resistome patterns in AWW primarily reflect aggregated fecal-associated bacterial communities carrying transferable resistance and virulence genes.

## 4. Discussion

This study evaluates AWW as a complementary environmental matrix for AMR surveillance by integrating high-throughput qPCR, shotgun metagenomic sequencing, and genome-resolved metagenomic analysis. In contrast to municipal wastewater, which reflects geographically fixed catchments and relatively stable populations, AWW aggregates microbial and genetic material from highly mobile, internationally diverse populations over short temporal windows. When analyzed systematically, this matrix offers a spatially compressed, mobility-linked surveillance perspective that can complement established wastewater-based and clinical surveillance systems rather than replace them [18,19]. It also provides a window for monitoring the introduction of AMR signals, particularly within major global travel hubs. Previous studies have demonstrated the feasibility of using aircraft and airport-associated wastewater for pathogen surveillance, particularly for viral detection during the COVID-19 pandemic [6,19]. More recent work has explored the resistome of AWW, demonstrating both the presence of clinically relevant ARGs and compositional differences between aircraft and terminal wastewater [3,9]. These studies have largely employed qPCR or metagenomic quantification without integrating a multi-layered genomics framework linking ARGs to microbial community members at genome-level resolution [3,9].

In this study, high-throughput qPCR profiling demonstrated consistently high detection rates of taxonomic markers, ARGs, and MGEs across all AWW samples, with 57–65 of 72 targets detected per sample. The widespread presence of resistance determinants spanning multiple antimicrobial classes across all flight origins indicates a shared baseline resistome reflective of globally circulating resistance reservoirs. Comparable baseline signals have been reported in municipal wastewater surveillance across diverse geographic contexts, where aggregated human shedding integrates heterogeneous antimicrobial exposure histories and microbiomes [4,20,21]. In this respect, AWW captures a resistome signal shaped primarily by international mobility rather than local catchment structure. Importantly, within this shared resistome background, qPCR analysis revealed regional structuring in the relative abundance and occurrence of specific resistance genes and MGEs. The detection of carbapenemase markers (*bla*_IMP-1_, *bla*_KPC-2_, *bla*_NDM_) exclusively in wastewater from African and Asia-Pacific flights, alongside elevated relative abundances of selected β-lactamase genes in these cohorts, mirrors well-documented geographic heterogeneity in carbapenem resistance reported by global clinical and environmental surveillance systems [22,23]. These findings highlight the sensitivity of AWW for detecting geographically structured resistance signals at the population level, while recognizing that such signals reflect aggregated carriage patterns rather than prevalence, transmission dynamics, or disease burden.

In contrast to high-throughput qPCR, the use of shotgun metagenomic sequencing provided an untargeted, community-wide perspective that contextualized these targeted findings within the broader microbial ecology. Across the ten AWW metagenomes, high microbial diversity was observed, with 752 bacterial species detected and consistently elevated alpha diversity across samples. The dominance of gut-associated families such as *Coriobacteriaceae*, *Bifidobacteriaceae*, *Lachnospiraceae*, *Ruminococcaceae*, and *Streptococcaceae* strongly supports the interpretation that AWW primarily reflects aggregated intestinal microbiota from passengers and crew [2,3,9]. This taxonomic structure closely parallels that reported in large-scale human gut and municipal wastewater metagenomic studies, reinforcing the use of AWW for AMR surveillance. This will serve as an extension of established wastewater-based epidemiology rather than a distinct ecological niche. Indeed, similar alpha diversity across flight-origin regions suggests that AWW provides a relatively stable substrate for comparative analyses, despite expected variation in the relative abundance of individual taxa between flights. Such variation likely reflects differences in passenger demographics, diet, health status, antimicrobial exposure, and travel patterns. The consistent detection of *Enterobacterales*, including *E. coli*, *K. pneumoniae*, and *S. enterica*, further reflects their central role as contributors to the human-associated resistome in wastewater environments [24].

At the functional level, shotgun metagenomic analysis identified a diverse resistome comprising 345 ARGs, with statistically significant differences across flight-origin regions. The predominance of tetracycline resistance genes across all samples reflects sustained selective pressure arising from long-standing antimicrobial use in both clinical and agricultural contexts and represents a recurrent feature of global wastewater resistomes [4]. The concurrent detection of resistance determinants associated with macrolides, folate-pathway inhibitors, β-lactams, quinolones, colistin, and glycopeptides illustrates the breadth of resistance captured by untargeted sequencing. Despite the homogenizing influence of international air travel, geographic structuring of resistome profiles was observed, supporting the use of AWW for comparative trend detection and horizon scanning rather than prevalence estimation. Virulome profiling at the read level revealed widespread detection of VFGs associated with adhesion, biofilm formation, immune modulation, and iron acquisition. Many virulence-associated genes fulfil multifunctional roles in colonization, environmental persistence, and microbial competition, and their detection in wastewater does not equate to expression, pathogenicity, or transmission potential [25]. Regional variation in virulence gene profiles therefore most plausibly reflects population-level differences in strain composition and gene carriage rather than differences in clinical virulence or disease risk.

Genome-resolved metagenomic analysis provided a critical additional layer that functions both as validation and interpretive refinement of read-level findings. Reconstruction of 1012 MAGs, with over half meeting medium- or high-quality MIMAG criteria and stable proportions of high-quality MAGs across flight-origin regions, demonstrates that aircraft wastewater supports reproducible genome recovery despite its transient and composite nature. This observation aligns with findings from municipal wastewater and human gut metagenomes and indicates that read-level resistome and virulome signals are not artefacts of fragmented assemblies or compositional noise [26,27]. Beyond confirmation, MAG-based analysis adds interpretive value by enabling direct linkage of resistance and virulence determinants within individual genomes, thereby distinguishing true genomic co-occurrence from read-level co-detection arising from mixed community signals. Recovery of high- and medium-quality MAGs corresponding to *E. coli*, *E. faecium*, and *K. pneumoniae* demonstrates that AWW metagenomics can capture near-complete genomes of globally relevant taxa within an aggregated environmental matrix. Importantly, this genome-resolved perspective allows resistance gene content to be interpreted in a population-genomic context, strengthening confidence in regional resistome patterns observed using qPCR and read-level metagenomics and providing a robustness check against methodological biases inherent to individual analytical layers. MAG-based resistome analysis identified ARGs spanning multiple antimicrobial classes, including resistance to last-line agents, with dominance of glycopeptide resistance genes driven by *van* operon components. The frequent co-occurrence of multiple *van* genes within individual MAGs supports the presence of intact resistance operons at the population level rather than sporadic gene fragments, in keeping with enterococcal population structure in wastewater environments [28]. Regional variation in β-lactam, macrolide–lincosamide–streptogramin, tetracycline, and multidrug resistance-associated ARGs mirrored patterns observed in qPCR and read-level analyses, reinforcing the internal consistency and analytical robustness of the integrated framework. MAG-resolved virulome profiling further demonstrated geographic heterogeneity in virulence gene carriage. However, as with read-level virulome findings, these patterns are best interpreted as differences in gene carriage among circulating populations rather than indicators of pathogenic potential or clinical severity. Many of the virulence-associated genes detected encode multifunctional traits involved in colonization, persistence, and stress response, and their recovery within MAGs provides ecological context for community-level virulome profiles rather than evidence of enhanced virulence. Collectively, genome-resolved metagenomic analysis strengthens the interpretation of AWW surveillance by anchoring community-level resistance and virulence signals within taxonomically resolved genomes. Such host-resolved information is epidemiologically relevant because the significance of an ARG depends substantially on the organism carrying it. In a One Health context, this approach enhances the value of AWW as a sentinel matrix for comparing resistance gene–host associations across flight-associated populations and could complement clinical and conventional wastewater surveillance by providing greater taxonomic and genomic resolution.

Several limitations should be considered when interpreting these findings. First, AWW represents a composite and transient matrix influenced by passenger turnover, flight duration, onboard water use, as well as wastewater sanitation processes and retention times, which limit precise attribution of detected signals to specific populations or geographic locations. Second, variation in sequencing depth across samples influenced MAG recovery and detection sensitivity, as evidenced by reduced genome recovery in some cohorts. Third, each analytical layer carries inherent methodological biases related to primer selection, reference database completeness, assembly constraints, and annotation accuracy. Finally, AWW surveillance captures population-level carriage rather than clinical infection and should therefore be interpreted as a tool for early warning, comparative profiling, and trend monitoring rather than prevalence estimation or individual risk assessment. Against the backdrop of limited evidence in our setting, these findings provide important proof-of-concept support for the feasibility and potential value of this surveillance approach. However, given the short study duration and small sample size, larger longitudinal datasets with detailed flight metadata will be required to establish robust geographic signatures.

## 5. Conclusions

In conclusion, this study demonstrates that AWW provides a scalable, non-invasive environmental matrix capable of capturing microbial diversity, AMR reservoirs, virulence potential, and population-genomic structure associated with international travel. Within the context of intensifying global mobility, major international aviation hubs represent critical convergence points for microbial dissemination and environmental surveillance. For global hubs connecting multiple continents, AWW surveillance may be particularly valuable for early signal detection and comparative regional profiling of emerging resistance patterns, complementing established municipal wastewater and clinical surveillance systems [18,29]. From an environmental policy perspective, integration of AWW surveillance into AMR monitoring frameworks warrants further evaluation. Standardized sampling strategies, harmonized analytical pipelines, and cautious interpretation that clearly distinguishes surveillance signals from clinical inference will be essential. As international air travel continues to expand, AWW surveillance at major global hubs may represent a strategically relevant addition to the environmental surveillance landscape, supporting more mobility-aware and geographically informed monitoring of AMR.

## Figures and Tables

**Table 1:** Assembly and MAG recovery statistics across aircraft wastewater metagenomes.

Name	Cohort	Total Reads	Total MAGs	High-Quality MAGs N (%)	Medium-Quality MAGs N (%)	Mean Completeness (%)	Mean Contamination (%)
Aircraft_S1	Asia-Pacific	179,259,252	94	24 (25.5%)	25 (26.6%)	53.9	1.86
Aircraft_S2	Asia-Pacific	57,225,632	55	17 (30.9%)	19 (34.5%)	64.1	2.33
Aircraft_S3	Africa	264,108,264	168	35 (20.8%)	55 (32.7%)	57.0	2.61
Aircraft_S4	Europe	213,773,308	143	30 (21.0%)	49 (34.3%)	57.3	3.05
Aircraft_S5	North America	369,742,952	151	32 (21.2%)	49 (32.5%)	58.3	3.66
Aircraft_S6	Oceania	26,552,314	13	2 (15.4%)	4 (30.8%)	55.6	1.67
Aircraft_S7	South America	260,135,572	114	20 (17.5%)	41 (36.0%)	58.7	3.89
Aircraft_S8	South America	82,252,378	62	16 (25.8%)	24 (38.7%)	67.1	3.27
Aircraft_S9	Asia-Pacific	228,744,736	120	26 (21.7%)	38 (31.7%)	60.0	2.82
Aircraft_S10	Africa	78,630,184	92	20 (21.7%)	38 (41.3%)	62.7	2.72

Metagenome assembly and MAG recovery statistics for ten aircraft wastewater samples stratified by flight-origin region. High-quality MAGs (≥90% completeness, ≤5% contamination) and medium-quality MAGs (≥50% completeness, ≤10% contamination) are reported as absolute counts and percentages relative to the total number of MAGs per sample, following MIMAG standards.

## Data Availability

The sequencing data generated in this study have been deposited in the NCBI under BioProject accession PRJNA1511382.

## Supplementary Materials

The following supporting information can be downloaded at: https://www.mdpi.com/article/10.3390/microorganisms14091988/s1, Data S1: Gene targets included in the HT-qPCR panel adapted from the Resistomap One Health panel, with gene names, functional categories, and primer sequences; Table S1: Aircraft wastewater sample metadata and sequencing read counts; Table S2: Summary and quality of MAGs recovered by geographic region; Data S2: Antimicrobial resistance genes detected in MAGs and their predicted bacterial hosts.

## Abbreviations

The following abbreviations are used in this manuscript:

ABRicate	Antibiotic Resistance gene Identifier using BLAST-based annotation
AMR	Antimicrobial resistance
ARG	Antimicrobial resistance gene
AWW	Aircraft wastewater
bp	Base pair
CARD	Comprehensive Antibiotic Resistance Database
Ct	Cycle threshold
DNA	Deoxyribonucleic acid
GTDB	Genome Taxonomy Database
GTDB-Tk	Genome Taxonomy Database Toolkit
HT-qPCR	High-throughput quantitative polymerase chain reaction
MAG	Metagenome-assembled genome
MGE	Mobile genetic element
MIMAG	Minimum Information about a metagenome-assembled genome
MLS	Macrolide–lincosamide–streptogramin
MLSB	Macrolide–lincosamide–streptogramin B
PCoA	Principal coordinates analysis
PCA	Principal component analysis
PCR	Polymerase chain reaction
PERMANOVA	Permutational multivariate analysis of variance
qPCR	Quantitative polymerase chain reaction
RA	Relative abundance
RGI	Resistance Gene Identifier
rRNA	Ribosomal ribonucleic acid
SD	Standard deviation
UAE	United Arab Emirates
VF	Virulence factor
VFDB	Virulence Factor Database
VFG	Virulence factor gene
WBS	Wastewater-based surveillance
