# Supplementary material for: Aircraft Wastewater as a Sentinel for Transboundary Antimicrobial Resistance: An Integrated Genomic Approach
Source: Microorganisms. 2026 Sep 8;14(9):1988. doi: 10.3390/microorganisms14091988 (PMC13608942; doi:10.3390/microorganisms14091988)
Supplement: Supplementary file 1 [file microorganisms-14-01988-s001.zip › Supplementary Tables.pdf]

Table S1: Aircraft wastewater sample metadata and sequencing read counts.

Sample_ID	Region	Month	Year	Reads Total
Aircraft_S1	Asia-Pacific	October	2024	179259252
Aircraft_S2	Asia-Pacific	October	2024	57225632
Aircraft_S3	Africa	November	2024	264108264
Aircraft_S4	Europe	November	2024	213773308
Aircraft_S5	North America	December	2024	369742952
Aircraft_S6	Oceania	December	2024	26552314
Aircraft_S7	South America	January	2025	260135572
Aircraft_S8	South America	January	2025	82252378
Aircraft_S9	Asia-Pacific	Febuary	2025	228744736
Aircraft_S10	Africa	Febuary	2025	78630184

Table S2: Summary and quality of MAGs recovered by geographic region.

Region	No. of samples	Total MAGs	High-quality MAGs n (%)	Medium-quality MAGs n (%)	HQ+MQ MAGs n (%)
Africa	2	260	55 (21.2%)	93 (35.8%)	148 (56.9%)
Asia-Pacific	3	269	67 (24.9%)	82 (30.5%)	149 (55.4%)
Europe	1	143	30 (21.0%)	49 (34.3%)	79 (55.2%)
North America	1	151	32 (21.2%)	49 (32.5%)	81 (53.6%)
South America	2	176	36 (20.5%)	65 (36.9%)	101 (57.4%)
Oceania	1	13	2 (15.4%)	4 (30.8%)	6 (46.2%)
**Overall**	**10**	**1,012**	**222 (21.9%)**	**342 (33.8%)**	**564 (55.7%)**

Dubai Health - Internal Use
